# The Metabolic Potential of the Human Lung Microbiome

**DOI:** 10.3390/microorganisms12071448

**Published:** 2024-07-17

**Authors:** Florian Semmler, Matheus Regis Belisário-Ferrari, Maria Kulosa, Leonard Kaysser

**Affiliations:** Department of Pharmaceutical Biology, Institute for Drug Discovery, University of Leipzig, 04317 Leipzig, Germany; florian.semmler@medizin.uni-leipzig.de (F.S.); matheus.ferrari@uni-leipzig.de (M.R.B.-F.); maria.kulosa@medizin.uni-leipzig.de (M.K.)

**Keywords:** genome mining, metabolic potential, lung microbiome, biosynthetic gene cluster, mycobiome, bacterial interaction

## Abstract

The human lung microbiome remains largely underexplored, despite its potential implications in the pharmacokinetics of inhaled drugs and its involvement in lung diseases. Interactions within these bacterial communities and with the host are complex processes which often involve microbial small molecules. In this study, we employed a computational approach to describe the metabolic potential of the human lung microbiome. By utilizing antiSMASH and BiG-SCAPE software, we identified 1831 biosynthetic gene clusters for the production of specialized metabolites in a carefully compiled genome database of lung-associated bacteria and fungi. It was shown that RiPPs represent the largest class of natural products within the bacteriome, while NRPs constitute the largest class of natural products in the lung mycobiome. All predicted BGCs were further categorized into 767 gene cluster families, and a subsequent network analysis highlighted that these families are widely distributed and contain many uncharacterized members. Moreover, in-depth annotation allowed the assignment of certain gene clusters to putative lung-specific functions within the microbiome, such as osmoadaptation or surfactant synthesis. This study establishes the lung microbiome as a prolific source for secondary metabolites and lays the groundwork for detailed investigation of this unique environment.

## 1. Introduction

The lower airways are characterized by a warm and moist environment and provide attractive conditions for colonization by microbial communities. They are in constant contact with their aerial surroundings and in close proximity to the microbe-rich nasal and oral cavities, which ensures continuous exposure to different microorganisms. Notably, it has long been believed that the human lungs are sterile [1]. In recent years though, culture-independent molecular analyses identified a significant diversity of microorganisms in the lower respiratory tract, which previous approaches failed to detect [1].

Since then, the lung microbiome has gained increasing attention from the scientific community, but it is still vastly underexplored compared to the more established gastrointestinal and skin microbiomes [2]. This is partly due to the technical challenges in isolating and studying the low biomass and complex microbial environment of the lower respiratory tract [2]. However, understanding the nature and role of the lung microbiome in health and disease is an important element for developing personalized diagnostic and therapeutic approaches [3].

The lung microbiome, with its transient nature and dynamic composition significantly differs in both size and stability from the more consistent and densely populated microbiomes of regions, such as the skin, mouth, and gut. This difference is reflected in its notably lower microbial biomass of 10^3^–10^5^ bacteria per gram of tissue compared to 10^11^–10^12^ bacteria per gram of tissue in the gastrointestinal tract [1,4,5,6]. Some studies suggest that the lung microbiome originates from microaspirations of oral and nasal microbiotas [1,3]. However, other studies, including recent species-level evaluations, have identified distinct compositional differences between the lung and oral microbiomes. These findings indicate unique features and adaptation mechanisms in the lung microbial community [7,8,9,10].

The lung bacteriome of the healthy individuals is predominantly populated by species of Bacillota, Bacteroidota, Pseudomonadota, Fusobacteriota, and Actinomycetota, with *Prevotella*, *Streptococcus*, and *Veillonella* being the most prevalent genera [11,12] This diversity is dynamic and is influenced by environmental factors and health conditions [6,12]. The human lung mycobiome mainly consists of the phyla Eurotiomycetes, Saccharomycetes, Dothideomycetes, and Agaricomycetes. Some important genera within these phyla include *Aspergillus*, *Candida*, *Cladosporium*, *Hyphodontia*, and *Penicillium* [13,14].

Given its unique properties, the lung microbiome could serve as an excellent platform to study microbial interplay and the role of specialized metabolites. This regulation involves highly coordinated mechanisms processing, for example, communication, the secretion of bioactive compounds, and the formation of biofilms, all supporting the resilience and stability of the communities [15]. Natural products are often produced in this context as extracellular signaling molecules, which can modulate the expression of specific genes, sometimes beyond the species level [16,17]. As bioactive molecules, they can also establish colonization, suppress certain immune responses of the host, and shape microbial consortia by their selective toxicity [16,17]. Recently, human microbiomes have, thus, been explored as a source of bioactive small molecules to feed the drug development pipeline [18,19,20,21].

The human microbiomes of the gut, nose, and oral cavity have already been extensively investigated [22,23,24,25]. For instance, the chemical potential of the gut microbiome has been mapped in detail, showing a vast network of secondary metabolite BGCs, crucial for understanding microbial ecology and therapeutic applications [25]. Similarly, the BGCs from the oral microbiome, as explored by Aleti et al. 2019, highlight the diverse bacterial communication strategies involved in health and disease [23]. In contrast, the metabolic landscape of the lung microbiome remains completely undescribed. For that matter, microbiomes are not only locally influential, but affect distant organs and systems, as documented by interactions between the human lungs and the digestive tract or the brain [26,27]. While the interplay between the gut microbiome and host is well-documented [28,29], studies specifically targeting the lung microbiome in this context are still limited and demand more investigation.

In this study, we explore the metabolic potential of the human lung microbiome using a systematic computational approach [22,23,24,25]. Therefore, we compiled a specific database of 320 microorganisms associated with the human lung using the expanded Human Oral Microbiome Database V3.1 (eHOMD) [30] and the current literature. This allowed us to identify biosynthetic gene clusters (BGCs) using the antiSMASH software [31] and to classify them into gene cluster families (GCFs) by the BiG-SCAPE algorithm [32]. A similarity network of all the predicted BGCs revealed for the first time the vast metabolic landscape of the lung microbiome with distinct GCFs that could be assigned to putative lung-specific functions.

## 2. Materials and Methods

### 2.1. Computational Analysis of the BGCs from the Human Lung Microbiome

The database of genome sequences from lung-associated microbes was initially created using the work of Ibironke et al. 2020 and then expanded to include bacteria and fungi using the current literature and medical case reports [10]. The reference genomes of the listed species were obtained from the National Center of Biotechnology Information genome database. If complete genome sequences were not available, corresponding shotgun sequences were used. If no genomic sequence data were available at the time of analysis, the organisms were still retained in the list and included in the construction of the phylogenetic trees. The genome sequences were then analyzed for possible biosynthetic gene clusters using Antibiotics and Secondary Metabolite Analysis Shell, version 7.0 (antiSMASH) [31]. GraphPad Prism 9 software was used to visualize the counted BGCs.

### 2.2. Network Analysis

The GenBank files obtained from the antiSMASH output were processed using the Biosynthetic Gene Similarity Clustering and Prospecting Engine (BiG-SCAPE) software, version 1.1.5 [32]. Thereby, the respective gene clusters were grouped into common BGC families by similarity. Different similarity cutoff values of 0.3, 0.4, 0.6, and 0.8 were applied for this purpose. In addition, the “mix” setting was selected to generate a network including all BGC classes. Clustering with a cutoff of 0.8 showed adequate separation of the BGC families. Cytoscape 3.10.1 [33] software was used to visualize the generated networks. Using the Minimum Information about a Biosynthetic Gene cluster (MIBiG) database [34] and the BiG-SCAPE algorithm, the BGCs could be assigned to already known gene clusters.

### 2.3. Gene Cluster Comparisons

Gene cluster comparison figures were generated using the clinker workflow [35] to align and visualize the similarities of interesting gene clusters of the network with each other and with the MIBiG reference gene clusters. The GenBank output files from antiSMASH were used as the input for this. The default settings were applied for the clinker creation.

### 2.4. Phylogenetic Analysis 

Molecular Evolutionary Genetics Analysis (MEGA) software version 11 [36] was used to conduct phylogenetic analyses. First, the 16S rRNA and 28S rRNA sequences were obtained from the NCBI database. Then, two separate multiple sequence alignments were performed for the bacterial and fungal sequences using the MUSCLE algorithm. The phylogenetic trees were constructed with the neighbor-koining method using the p-distance model and by implementing a bootstrap test of 1000 replicates.

Finally, the web-based software Interactive Tree Of Life (iTOL) version 6.8.1 [37] was used to display the phylogenetic relationship of the organisms together with the determined BGCs.

## 3. Results

### 3.1. Compilation of a Unique Genome Database of Lung-Associated Microbes

To create a robust basis for our bioinformatic analyses, we compiled a specific database of genome sequences from lung-associated microbes (see Tables S1–S3). Therefore, we mainly relied on the work of Ibironke et al. 2020, which encompasses the most detailed catalog of bacteria within the healthy lung microbiome at the species level [10]. Our collection was further expanded to include diverse bacterial and fungal species that were previously isolated from the human lung. This approach involved a careful examination of organisms listed in the expanded Human Oral Microbiome Database V3.1 (eHOMD), cross-referenced with recent publications and medical case reports to determine their occurrence in the airways. For fungi, we relied on recent publications describing the mycobiome of the human lung (see Table S2). In total, we included 227 bacterial strains and 93 fungi to build a first representative database of the microbiome of the human lower respiratory tract.

### 3.2. The Human Lung Microbiome Contains a Variety of Biosynthetic Gene Clusters across Phylogenetic Boundaries

**Figure 1:**
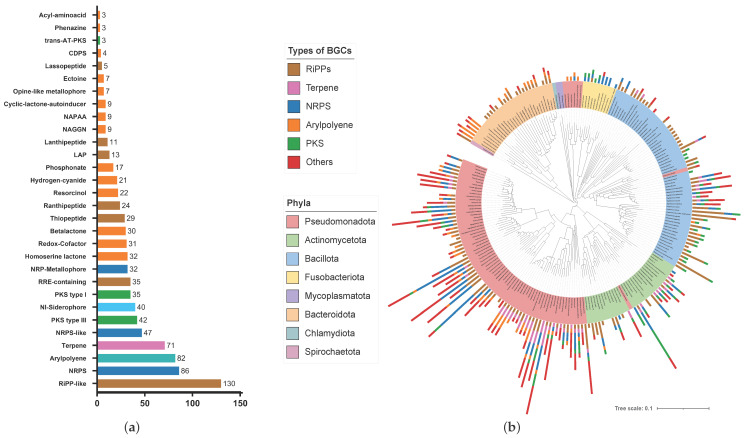
The distribution of biosynthetic gene clusters of bacteria in the human lung microbiome. (**a**) Bar chart showing the number of respective subclasses of gene clusters of all bacteria studied; (**b**) phylogenetic tree representing the evolutionary relationship of the bacteria in combination with the distribution of biosynthetic pathways for natural products.

**Figure 2:**
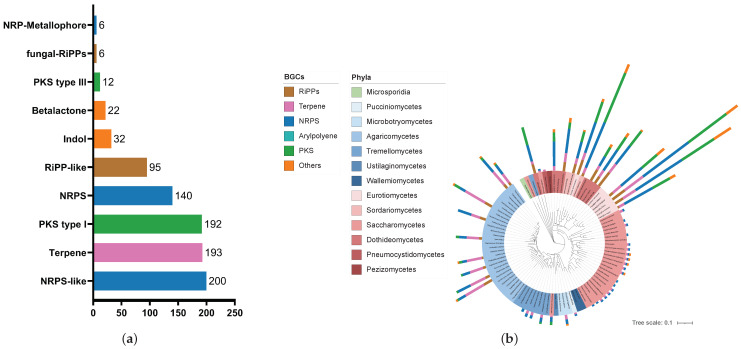
The distribution of biosynthetic gene clusters of fungi in the human lung mycobiome. (**a**) Bar chart showing the number of respective subclasses of gene clusters of all fungi studied; (**b**) phylogenetic tree representing the relatedness of the fungi in combination with the distribution of gene clusters as higher natural product classes.

Subsequently, we employed established computational genome mining techniques to gain insights into the natural product pathways carried by these microorganisms. Using the antiSMASH 7.0 platform [31], a total of 1832 biosynthetic gene clusters (BGCs), comprising 917 pathways from bacteria and 914 pathways from fungi were identified. These clusters were further categorized by natural product classes, e.g., non-ribosomal peptides (NRPs), terpenes, polyketides (PK), ribosomally synthesized and post-translationally modified peptides (RiPPs) and aryl polyenes. On average, bacteria feature four BGCs per organism, with a predicted overall metabolic diversity of 28% RiPPs, 18% NRPs, 9%, arylpolyenes, 9% PKs and 8% terpenes (see Figure 1). The remaining 28% belong to other classes of natural products, such as various types of metallophores (8.8%), homoserine lactones (3.5%), redox cofactors (3.4%), betalactones (3.3%), and resorcinols (2.4%). In contrast, fungi have on average almost ten BGCs per organism, being 38% NRPs, 23% PKs, 21% terpenes, 10% RiPP-like, 1% RiPPs, and 7% others (see Figure 2).

In our analysis, the Pseudomonadota exhibit the highest abundance of biosynthetic gene clusters among bacteria, with a total count of 537 BGCs and an average of 6.8 BGCs per organism (Table 1). Further investigations of this phylum showed that species of *Pseudomonas*, *Paraburkholderia*, and *Microbulbifer* feature a particularly rich specialized metabolism, with 13.6, 15.7, and 12.5 BGCs per organism, respectively. The phylum of Bacillota accounts for 194 of the BGCs, but with 2.9 BGCs per organism on average, the typical member of this phylum cannot be considered as particularly natural-product proficient. This observation, though, may be influenced by the number of sampled organisms or the size of their genomes. Nevertheless, recent investigations have described various human-associated *Staphylococcus* and *Streptococcus* species as metabolically versatile [38,39,40,41]. Actually, with a respective 4.4 and 6.5 BGCs per organism on average, these genera may also play a role as natural product producers in the human lung. Another BGC rich group of bacteria include several representatives of the genus *Mycobacterium* from the phylum Actinomycetota, with an average of 11.5 BGCs per organism. Mycobacteria are known to exercise a diverse repertoire of metabolic capacities [42].

Among the fungi, the Ascomycota (14.7) have substantially more BGCs per organism than the Basidomycota (4.9). Within the Ascomycota group, there are large differences in the distribution of gene clusters among the various phyla. For example, the Eurotiomycetes, Dothideomycetes, and Sordariomycetes have a particularly high number of gene clusters with a total of 334, 205, and 108 BGCs, respectively. Some genera, such as *Aspergillus*, *Alternaria*, and *Bionectria*, show a particularly extensive secondary metabolism with 70.5, 38.5, and 79 BGCs per organism, respectively. Filamentous fungi of the genus *Aspergillus* are known for their metabolic capacities and, therefore, have a wide range of biotechnological applications [43,44,45,46]. Various secondary metabolites have already been described from Alternaria and Bionectria species [47,48,49]. Among the Basidomycota, representatives of the Agaricomycetes phylum, such as *Trametes* spp., *Piptoporus betulinus*, *Pleurotus pulmonarius*, and *Plicaturopsis crispa*, contain a particularly high number of BGCs.

### 3.3. Correlation between Genome Size and BGC Count

**Figure 3:**
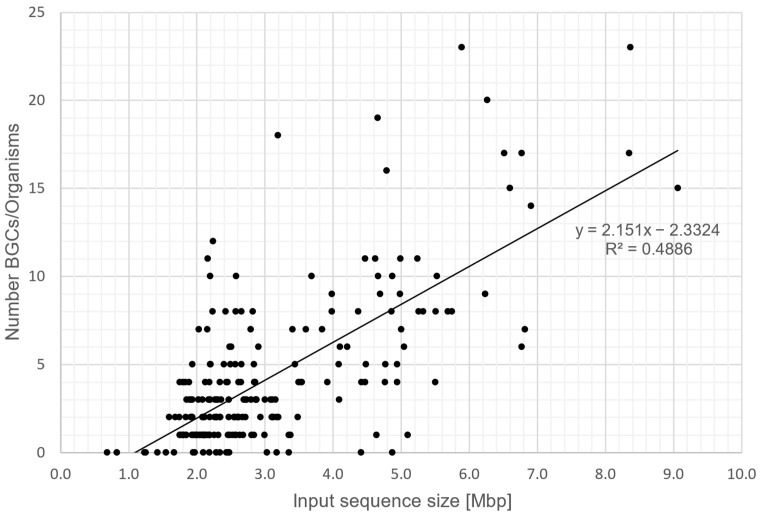
Input sequence size plotted against the number of predicted BGCs in bacteria of the human lung microbiome. Each dot corresponds to one bacterial strain of our database. Trendline indicates a positive correlation between the number of BGCs present in a strain and its input sequence size (coefficient of determination (R^2^ = 0.4886)).

To evaluate the correlation between genome size and BGC count in the lung microbiome, we plotted the average sequence size against the number of BGCs per organism (Figure 3). Our analysis indicates a positive correlation between these two traits. This is in line with studies by Cimermancic et al. 2014 (R2 = 0.56) and Stubbendieck et al. 2021 (τB = 0.35), which observed a similarly strong correlation between these two variables [50,51]. Similar to other specific ecological niches, the lung microbiome appears to be inhabited by highly adaptable generalists with large genomes and numerous different pathways for diverse ecological situations. On the other hand, there are small-genome specialists that are highly adapted to their host and which have likely lost most of the non-essential genetic information in the co-evolution process.

### 3.4. A Similarity Network of Gene Cluster Families Reveals a Multitude of New Putative Secondary Metabolites

**Figure 4:**
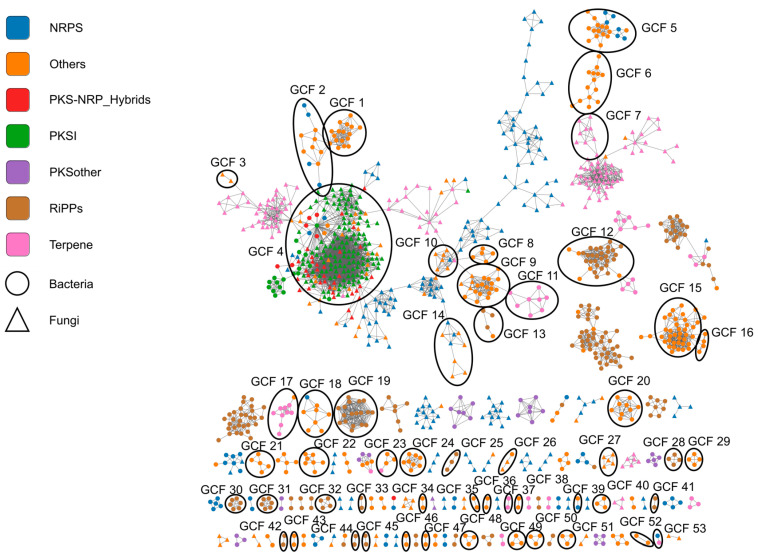
BiG-SCAPE sequence similarity network (SSN) of the identified biosynthetic gene clusters of all the investigated organisms of the human lung microbiome. The network files used were obtained after BiG-SCAPE analysis with a cutoff value of 0.8 and the mix parameter. Subsequent visualization was performed using Cytoscape. Different colors represent classes of natural products; shapes indicate the domain of life of the organism. Important groups of GCFs that are discussed here are marked with a frame.

A sequence similarity network (SSN) was generated using the BiG-SCAPE algorithm to investigate the detected gene clusters and their structural relationship. The resulting SSN consists of 1154 nodes with 4268 connecting edges (see Figure 4). The separation of homologous gene clusters into distinct families was achieved with a cutoff value of 0.8. Therefore, a total of 767 of these gene cluster families (GCFs) consisting of at least two different BGCs and 520 singletons were identified (see Table S4). The annotation of the GCFs was additionally informed by the Minimum Information about a Biosynthetic Gene cluster (MIBiG) database [34].

At the center of the network is an extensive supercluster, dominated by diverse type I PKS pathways from fungi. Similar to NRPs, type I polyketides are produced via large, modular, multi-enzymatic megasynthases. They are primarily found in bacteria and fungi, but have also been reported from algae, sponges, and mollusks [52,53]. Whereas type I PKS in bacteria mostly consist of linear multi-modular assembly lines, in fungi the majority of such systems are mono-modular and iterative. In both cases, an immense structural diversity is produced, correlating to a similar variety of bioactivities. In contrast, a number of other GCFs are considerably better defined and allow functional predictions. Two GCFs are predicted to encode for so-called compatible solutes, namely GCF 8 for ectoin-like compounds and GCF 24 for N-acetylglutaminylglutamine amide (NAGGN) derivatives. Furthermore, a variety of different GCFs with putative antioxidant function can be found, such as GCFs 12 and 45 as analogs to the peptide redox cofactor pyrroloquinoline quinone (PQQ), GCF 7, 11, 17, 37, and 53 with similarities to carotenoid gene clusters, and potential aryl polyenes in GCF 15, 20, 34, 40, 46, 47, 51, and 52. Various GCFs could be assigned to natural product classes with putative antimicrobial functions, such as antimicrobial RiPPs (GCF 3, 9, 10, 19, 27, 29, 32, 33, 41, 42, and 44), betalactones (GCF 3, 9, 10, 27, and 29), and hydrogen cyanides (GCF 1). Putative pathways for communication in the lung microbiome were found in gene clusters of GCF 16, 21, 38, 49, and 50 as N-acyl homoserine lactones and in GCF 30 as cyclic lactone autoinducers. In addition, it was predicted that BGCs of GCF 2, 9, and 10 encode for natural products that could be related to surfactant synthesis. Finally, a number of GCFs, such as 2, 5, 6, 14, 18, 22, 23, 26, 35, 36, and 43, were also identified in our analysis as containing potential metal chelator pathways.

## 4. Discussion

The aim of the study is to provide a first description of the metabolic landscape of the human lung microbiome in order to establish the basis for the investigation of undescribed gene clusters, and also to find the association of gene cluster families with functions within this microbial community. One of the first systematic studies of this kind was carried out by Cimermancic et al., who searched for putative BGCs in 1154 sequenced genomes from all bacterial phyla in order to create a global map of the biosynthetic universe [50]. Here, too, the Pseudomonadota represented the largest phylum in the analysis, which was particularly characterized by the widespread distribution of different groups of aryl polyenes. Our analysis showed that the Pseudomondota also dominate the bacteriome of the lung. However, we found RiPPs to be the most abundant type of pathways. This may be explained by the fact that RiPPs have been extensively investigated in the last ten years and were given a universal nomenclature by Arnison et al. only in 2013 [54].

In a recent study, Aleti et al. explored the metabolic potential of the oral microbiome [23]. Because the lung microbiome is considered to mainly originate from the microbial communities of the upper airways, it is worth comparing the results from Aleti et al. with our data. Notably, the most diverse phyla in terms of BGCs are consistent in both the oral and the lung microbiome, including Pseudomonadota, Bacillota, Actinomycetota, and Bacteroidota, respectively. Furthermore, the distribution of BGCs across these groups also demonstrated similarities. For example, RiPPs emerged as the most abundant BGC type in the oral microbiome, followed by NRPS and aryl polyenes, which matches the hierarchy identified by our analysis of the lung microbiome. These shared characteristics lend support to the hypothesis of microaspiration as a major driving force behind lung microbiome development. However, a study by Stubbendieck et al., which investigated the specialized metabolism of the aerodigestive tract microbiome, revealed significant differences both in the composition of the respective microbiota and in the distribution of the corresponding BGCs [51]. This distinct biogeography of the upper respiratory tract underlines the need for a closer examination of the microbiome of the lower respiratory tract and the lungs with regard to its metabolic potential.

### 4.1. BGC Distribution and Metabolic Diversity

In general, our analysis of the lung mycobiome shows a two-part image in respect of chemical capacity. A few organisms are very rich in BGCs, while the majority carry only a small number or no pathways for the common types of secondary metabolites. In addition, the natural product classes are differently distributed in fungi compared to in bacteria. In our analysis, NRPs, PKs, and terpenes are much more frequent, whereas RiPPs appear to play a minor role in fungi. However, this result may be biased because these compounds have not been investigated in fungi for very long. In 2007, the elucidation of the biosynthetic pathway of amanitin marked the first RiPP of fungal origin [55]. Since then, only four subclasses of fungal RiPPs have been characterized. Although many of these RiPPs, such as amatoxins or phallotoxins, show toxic effects to humans, their genuine ecological functions are not known [56].

The high abundance of BGCs in Pseudomonadota, especially in species of *Pseudomonas*, *Paraburkholderia*, and *Microbulbifer*, aligns with their known adaptability to nutrient-poor and challenging environments [57,58,59,60,61,62]. Their diverse secondary metabolism likely contributes to their robust biological defense mechanisms, enabling them to outcompete or inhibit the growth of microbial contenders. In fungi, the significant metabolic capacities of Eurotiomycetes, Dothideomycetes, and Sordariomycetes, as exemplified by genera, like *Aspergillus*, *Alternaria*, and *Bionectria*, reflect their wide range of biotechnological applications. The presence of numerous BGCs in these groups underscores their potential as prolific sources of natural products.

### 4.2. Assignment of Putative Pathways for Osmoadaptation

In our analysis, two different types of pathways were predicted to be responsible for the production of so-called compatible solutes, i.e., the ectoin-like and N-acetylglutaminylglutamine amide (NAGGN)-like BGCs. In the SSN, the ectoin cluster is found in GCF 8 with five nodes and in the NAGGN cluster as GCF 24 with eight nodes (Figure 4). Here, both of them are only present in organisms of the Pseudomonadota and are particularly widespread within the genus *Pseudomonas*. Both compound classes have been described as osmoprotective secondary metabolites [63,64,65,66]. When microorganisms colonize the human lung, they are confronted with the constantly changing osmotic conditions accompanied by the human respiratory cycle [67]. For example, it has been known for a long time that exercise-induced hyperventilation can lead to hyperosmolar conditions in the airways [68]. It is, therefore, important for lung microbiota to have effective systems to cope with high osmolarities [69]. This osmoadaptation can be mediated by the synthesis of osmoprotective substances, such as ectoins and NAGGN, which prevent swelling and cell lysis under these conditions [70,71]. Some of these osmoprotective metabolites have also been associated with virulence factors in pathogenic bacteria [70].

### 4.3. Assignment of Putative Pathways for Antioxidants

The lung promotes gas exchange for the entire human body and is, thus, especially susceptible to oxidative stress. This can be facilitated by external exposures, such as air pollution, or internal mechanisms, as in inflammation. As a countermeasure, bacteria and fungi may produce small-molecule antioxidants to protect themselves from reactive oxygen species that are typically abundant in such situations. Notably, our analyses predicted 29 gene clusters that produce compounds similar to the peptide redox cofactor pyrroloquinoline quinone (PQQ) [72], summarized in GCF 12 and GCF 45. This peptide is important in the methylotrophic metabolism and ethanol/glucose utilization in proteobacteria and few others [72]. In our analysis the corresponding BGCs are mainly found in Pseudomonadota, such as *Pseudomonas*, *Paraburkholderia*, and *Enterobacter*, as well as specific representatives of the phylum Actinomycetota. These include some pathogenic species, such as *Pseudomonas aeruginosa*, *Serratia marcescens*, and *Mycobacterium tuberculosis*. The antioxidant effect of PQQ has been demonstrated in vivo. But PQQ has also been detected in mammalian and human tissue samples [73,74,75] and been described as a “longevity vitamin” for humans [76,77]. The production of PQQ by lung-associated bacteria might, thus, be beneficial for the host. Since some PQQ-producing bacteria establish symbiotic relationships with other microorganisms, the possible involvement of PQQ derivatives in the human lung in microbe-host interaction could be an interesting subject for further investigation [72].

Another group of antioxidants are small-molecule pigments e.g., carotenoid-type terpenoids and aryl polyene-type polyketides. With their extensively conjugated systems, such compounds are able to scavenge high-energy radicals and are particularly indispensable for photosynthetically active organisms [78]. However, their functions go much further, as they have been correlated with protection against extreme temperatures and UV radiation, antimicrobial activity, and the ability to acquire nutrients [79,80,81,82].

In our analysis, many of the 264 identified terpene-synthesizing pathways can be assigned to reference gene clusters of carotenoids, such as β-carotene or zeaxanthin (see Figure S1). They mostly originate from different Pseudomonadota, such as *Pseudomonas* spp. and *Pantoea* spp., *Novosphingobium panipatense* and *Paracoccus yeei*, but also from fungi, such as *Alternaria alternata*, *Cladosporium cladosporioides*, *Aspergillus flavus* and *Plectosphaerella cucumerina*. These BGCs cluster in GCFs 7, 11, 17, 37, and 53. Furthermore, BGCs which code for potential aryl polyenes are found in GCF 15, 20, 34, 40, 46, 47, 51, and 52 (see Figure S2). This group has similar antioxidant properties to the chemically related but biosynthetically distinct carotenoids [50]. In our SSN, these gene clusters originate from various *Prevotella*, *Paraburkholderia*, *Aggregatibacter*, and *Pseudomonas* species.

### 4.4. Assignment of Putative Pathways for Antimicrobials

Metabolites with antimicrobial activity play a decisive role in regulating the stability of the microbiome and its different subpopulations. They either act specifically against certain groups or non-specifically against many different species. Not surprisingly, various of these compounds can be found in our network. These include different classes of RiPPs, such as lanthipeptides (GCF 41 and 44), thiopeptides (GCF 19 and 32), and linear azole-containing peptides (LAP) (GCF 33 and 42) [83,84,85], but also some betalactone compounds (GCF 3, 9, 10, 27, and 29) [86,87,88,89,90].

An interesting example of highly targeted antimicrobial activity may be represented by a putative BGC from *Streptococcus intermedius*, that is homologous to the gene clusters of tryglysin from *Streptococcus mutans* and streptide from *Streptococcus thermophilus* (see Figure S3). These radical S-adenosylmethionine (RaS)-type RiPPs have been shown to exhibit growth-inhibitory activity against *Streptococcus* spp. but have also been studied in the context of intraspecies communication [91,92,93]. Their biosynthesis is regulated by a bona fide short hydrophobic peptide (SHP)/Rgg quorum-sensing mechanism, which is conserved throughout the genus *Streptococcus*. Consequently, it was demonstrated that tryglysin modulates specifically the growth of streptococci, but not other Gram-positive organisms [93]. Since the genus *Streptococcus* is an important group within the respiratory system, such antibacterial metabolites have the potential to be influential regulators of the lung microbiome. For example, by specifically inhibiting the growth of commensal streptococci, this imbalance could be exploited by non-streptococci pathogens. Or, vice versa, commensal *Streptococcus* spp. could suppress establishment of pathogenic streptococci at potential infection sites.

GCF 9 contains twenty different betalactone gene clusters. They derive from diverse genera, such as *Acinetobacter*, *Pseudomonas*, and *Aspergillus*. Betalactones exhibit a highly reactive four-membered heterocycle of an electrophilic nature [94]. This reactivity gives rise to a diverse range of possible biological properties [95], e.g., antimicrobial and anti-cancer activity [86]. But, generally, these molecules inhibit hydrolytic enzymes, including proteases and lipases, which are ubiquitously secreted into the lung surface liquid by human tissue cells and colonizing microorganisms. Whether betalactones could have a protective role in such an environment for commensal or pathogenic bacteria would be an interesting subject for further investigations. In general, analogous clusters have been identified in both bacterial and fungal contexts [95,96,97].

In our model, GCF 1 with 17 members can be assigned to the synthesis of hydrogen cyanide (HCN) by different species of the Pseudomonadota, such as *Burkholderia cepacia*, *Brucella anthropi*, *Achromobacter xylosoxidans*, and *Pseudomonas fluorescens*. It has recently been shown that the respiratory pathogen *Pseudomonas aeruginosa* [98] produces airborne volatile hydrogen cyanides and thereby inhibits the growth of *Staphylococcus aureus*. This is a particularly relevant mechanism in the prognosis of cystic fibrosis, as both bacteria are most prevalent in the mucus of patients with this lung disease [99,100]. The expression of HCN gene clusters is often dependent on the population density of *P. aeruginosa* and is controlled by quorum sensing [101]. By using cyanide-deficient mutants of *Pseudomonas* strains and subsequent transcriptome analyses in environmental samples, it could be shown that a variety of different cellular processes, such as their ability to take up iron and their swimming motility, are influenced by HCN both endogenously and exogenously [102]. Accordingly, there may be a multi-layered potential influence within the lung microbiome as well. Interestingly, GCF 1 includes mainly non-pathogenic species, opening speculations about a suppressive role of HCNs in the lung against the colonization of *Staphylococcus aureus*.

### 4.5. Assignment of Putative Pathways for Communication

A highly regulated coordination has been described for many essential processes within the microbiome, such as the formation of biofilms, sporulation, bioluminescence, toxin production, and swarming [103]. One of the most important communication mechanisms is quorum sensing, the population-dependent expression of target genes. Quorum sensing relies on the secretion of autoinducers, specialized secondary metabolites [104,105]. Different groups of bacteria apply different compounds as signaling molecules. The homoserine lactones are a group of autoinducers from Gram-negative bacteria which have been studied extensively, in particular the N-acyl homoserine lactones [106,107,108]. In our SSN, the 17 members of this group are organized in 5 different GCFs (16, 21, 38, 49, and 50), and derived from Pseudomonadota, for example from various species of *Neisseria*, *Paraburkholderia*, and *Pantoea*. In these GCFs, the BGCs are grouped together with other BGCs from their respective genus.

In contrast, Gram-positive bacteria usually use small peptides as autoinducer molecules [104]. One of these groups, the so-called cyclic lactone autoinducers, can also be found in our network. This group consists of six nodes that are summarized in GCF 30. These compounds usually consist of seven to nine amino acids and have a variable amino acid sequence [109]. However, a thiolactone ring consisting of five amino acids appears to be relevant for their function, which is present in all described specimens of this group [109]. The gene clusters have so far only been reported from *Staphylococcus* species, matching the results in our SSN.

### 4.6. Assignment of Putative Pathways for Surfactants

A number of different NRPS gene clusters were identified that are predicted to encode for lipopeptides with the ability to lower surface tension. These biosurfactants are secondary metabolites with similar biophysical properties to human pulmonary surfactant, and are involved in processes, such as swarming motility, biofilm formation, and chemical warfare [110,111]. In GCFs 2, 9, and 10, a total of 16 BGCs from the Pseudomonadota and Bacillota, such as *Bacillus altitudinis*, *Pseudomonas aeruginosa*, *Delftia acidovorans*, *Microbulbifer halophilus*, and *Bacillus anthracis*, are homologous to pathways from the known biosurfactant lipopeptides fengycin, surfactin, iturin, viscosin, or entolysin. Since these lipopeptides can also be found in fungi, BGCs from *Aspergillus flavus* could be assigned to the reference gene cluster of syringafactin and from *Aspergillus versicolor* to the reference gene cluster of fengycin (see Figure S4). The lung-associated microbial communities mostly reside within the extracellular mucus layer. Changes in the texture, viscosity, and other physicochemical characteristics of mucus have great effects on the accessibility and stability of this ecological niche and its inhabitants. It could be speculated that the organisms of the human lung microbiome form small-molecule biosurfactants to modulate mucus properties and, thus, facilitate colonization in this environment. Furthermore, the amphiphilic molecular properties of those lipopeptides can reduce the surface tension of the pulmonary liquid to impair host-derived defense mechanisms of the respiratory tract. Since both bacterial and fungal gene clusters with similarities to these lipopeptides are found, it is possible that they confer a potential survival advantage in the human lung.

### 4.7. Assignment of Putative Pathways for Metal Chelators

The acquisition of metal ions, which are structurally and catalytically integrated into many essential proteins, poses a particular challenge for microorganisms [112]. This task is, therefore, both part of interspecies competition and an opportunity for the host to influence the growth of the organisms through targeted retention of nutrients, so-called nutritional immunity [113,114]. Many microorganisms produce specialized small molecules that can bind metal ions from the environment with great affinity and selectivity and can then be reabsorbed by specific transporters [115]. Depending on the metals they bind, these metallophores are referred to as siderophores (Fe^3+^), zincophores (Zn^2+^), chalcophores (Cu^2+^), molybdophores (Mo^4+^/MoO_4_^2−^), or nickelophores (Ni^2+^) [116,117]. They are produced by both fungi and bacteria and are also relevant as pathogenicity factors due to their crucial role in metal homeostasis [118,119].

Consequently, our SSN analysis revealed a number of different metallophore gene clusters in the human lung microbiome. For example, we have found twelve putative zincophore gene clusters of the Cnt-type [112] in the phyla Pseudomonadota and Bacillota. Here, BGCs from *Staphylococcus* spp., *Brucella anthropi*, *Furfurilactobacillus rossiae*, *Serratia marcescens*, *Yersinia pestis*, *Pseudomonas aeruginosa*, and others show homology to certain representatives of zincophore pathways producing, e.g., bacillopalins, staphylopins, pseudopalins, or yersinopins (see Figure S5). In addition, we discovered numerous pathways for siderophores, which is the most studied group of metallophores, in GCFs 2, 5, 6, 14, 18, 22, 23, 26, 35, 36, and 43. According to their biosynthesis one can distinguish NRPS-independent siderophores, such as aerobactin from NRPS-dependent ones, such as enterobactin (see Figure S6). While most of the BGCs for the NRPS-independent metallophores are grouped in GCF 5, 6, 18, 22, 26, 35, and 43, the NRP-dependent metallophores can be found in GCF 2, 5, 14, 23, and 36. Ten BGCs show similarities to aerobactin, whereas twenty BGCs show similarities to enterobactin. One of the putative gene clusters for the synthesis of enterobactin was found in the Gram-positive bacterium *Rothia mucilogenosa*, also a known inhabitant of the human oral cavity [120. Notably, previous studies have established a link between *Rothia* species in the oral microbiome and the iron balance in this niche [120]. It has been shown that the enterobactin produced by *Rothia mucilogenosa* influences the availability of iron both for the human host and for other members of the microbiome. Moreover, this compound is able to inhibit the growth of cariogenic strains of *Streptococcus mutans*, commensal oral *Streptococcus* species, of methicillin-resistant strains of *Staphylococcus aureus* (MRSA) and oral *Actinomyces timonensis* [120]. A specific mechanism behind this observation is not yet known, but it, therefore, might be a potential therapeutic target for further investigation. 

## 5. Conclusions

Our study reveals the extensive metabolic potential of the human lung microbiome, highlighting a diverse network of biosynthetic gene clusters. We identified numerous previously undescribed BGCs, which likely play decisive roles in environmental adaptation, microbial competition, nutrient acquisition, and communication, underscoring the lung microbiome’s unique metabolic diversity. The discovery of these new BGCs suggests the lung microbiome as a promising source of novel natural products, which could be developed into therapeutic agents. Understanding these metabolic pathways provides a basis for future research into innovative treatments for respiratory diseases, targeting specific microbial interactions and metabolic functions. Moreover, our analysis opens the perspective for the usage of specific metabolites as biomarkers in diagnostics or prognosis for the success of therapy in the future.

## Figures and Tables

**Table 1:** Distribution of BGCs in relevant phyla of the lung microbiome.

Kingdom	Phylum/Division	Number of Organisms	Number of BGCs	BGCs/Organism	Average Sequence Input Size (Mbp)
Bacteria	Actinomycetota	31	101	3.3	2.68
	Bacillota	67	194	2.9	2.31
	Bacteroidota	33	58	1.8	3.17
	Chlamydiota	1	-	-	1.23
	Fusobacteriota	13	23	1.8	2.37
	Mycoplasmatota	2	0	0.0	0.82
	Pseudomonadota	79	537	6.8	4.04
	Spirochaetota	1	4	4.0	2.84
	Total	227	917	4.0	2.43
Fungi	Ascomycota	47	693	14.7	21.00
	Dothideomycetes	8	166	20.8	39.03
	Eurotiomycetes	7	334	47.7	32.25
	Saccharomycetes	26	39	1.5	12.97
	Sordariomycetes	3	108	36	36.5
	Basidomycota	45	220	4.9	38.36
	Agaricomycetes	29	195	6.7	42.71
	Rozellomycota	1	-	-	-
	Total	93	913	9.8	29.68

## Data Availability

All data are contained within the article and Supplemental Materials.

## Supplementary Materials

The following supporting information can be downloaded at: https://www.mdpi.com/article/10.3390/microorganisms12071448/s1, Figure S1: Gene cluster comparison of different exemplary terpene geneclusters from our analysis and MIBiG reference clusters; Figure S2: Gene cluster comparison of different exemplary arylpolyene geneclusters from our analysis; Figure S3: Gene cluster comparison of different RaS-RiPP geneclusters within the genus *Streptococcus* and MIBiG reference clusters; Figure S4: BiG-SCAPE sequence similarity network (SSN) of different lipopeptide geneclusters and MIBiG reference clusters; Figure S5: Gene cluster comparison of different zincophore geneclusters and MIBiG reference clusters; Figure S6: BiG-SCAPE sequence similarity network (SSN) of different siderophore geneclusters from the enterobactin group and MIBiG reference clusters; Table S1: List of human lung bacteriome; Table S2: References for the human lung mycobiome; Table S3: List of human lung mycobiome; Table S4: List of GCFs of the SSN of the human lung microbiome [121,122,123,124,125,126,127,128,129,130,131,132,133,134,135,136,137,138,139,140,141,142,143,144,145,146,147,148,149,150,151,152,153,154,155,156,157,158,159,160,161,162,163,164,165,166,167,168,169,170,171,172,173,174,175,176,177,178,179,180,181,182,183,184,185,186,187,188,189,190,191,192,193,194,195,196,197,198,199,200,201,202,203,204,205,206,207,208,209,210,211,212,213,214,215,216,217,218,219,220,221,222,223,224,225,226,227,228,229,230,231,232,233,234,235,236,237,238,239,240,241,242,243,244,245,246,247,248,249,250,251,252,253,254,255,256,257,258,259,260,261].
