# Supplementary material for: The Metabolic Potential of the Human Lung Microbiome
Source: Microorganisms. 2024 Jul 17;12(7):1448. doi: 10.3390/microorganisms12071448 (PMC11278768; doi:10.3390/microorganisms12071448)
Supplement: Supplementary file 1 [file microorganisms-12-01448-s001.zip › Supplementary Figures.pdf]

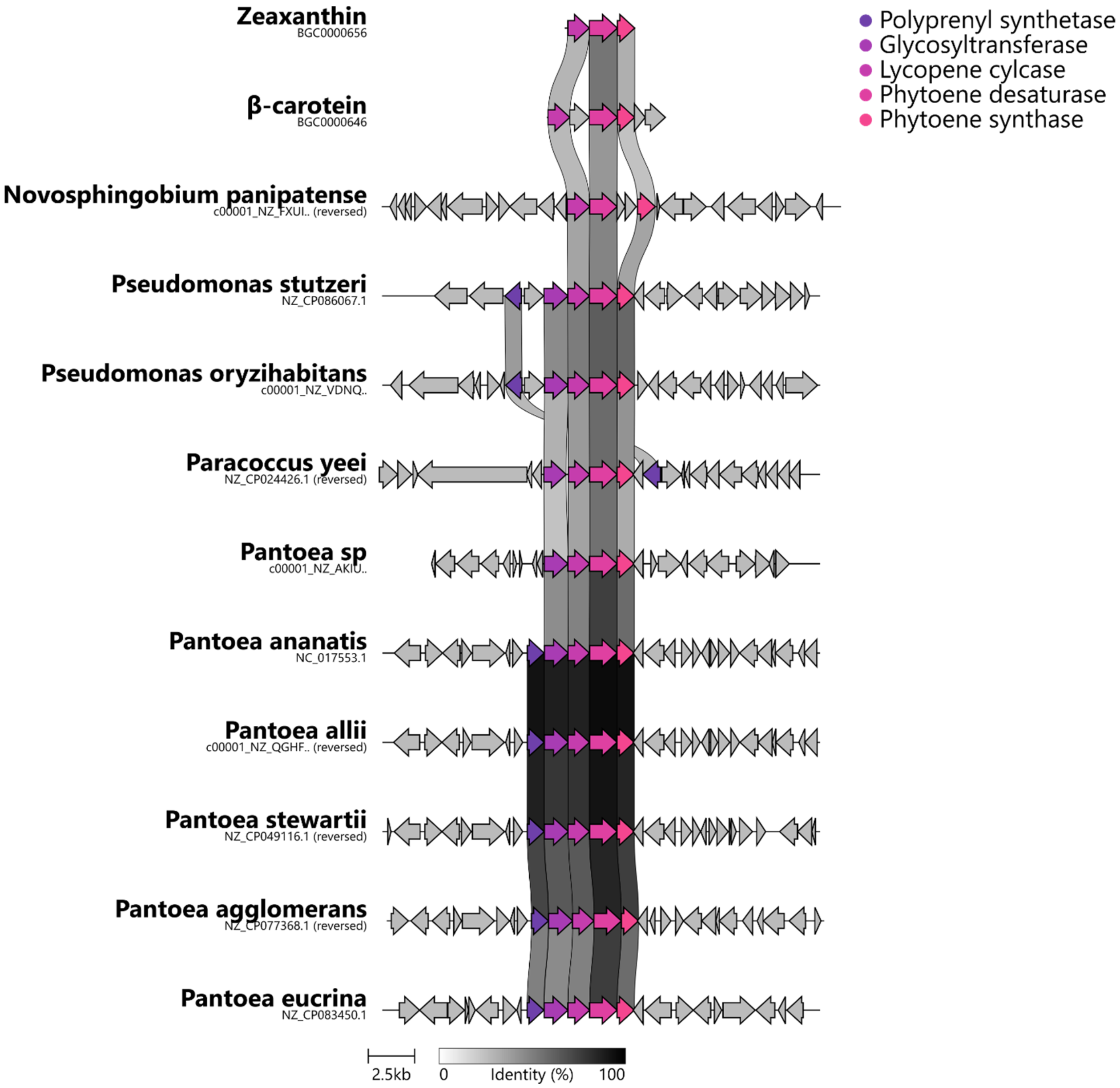

Figure S1: Gene cluster comparison of different exemplary terpene geneclusters from our analysis and MIBiG reference clusters. Genes within a gene cluster are color-coordinated. The links between the genes show their similiarity.

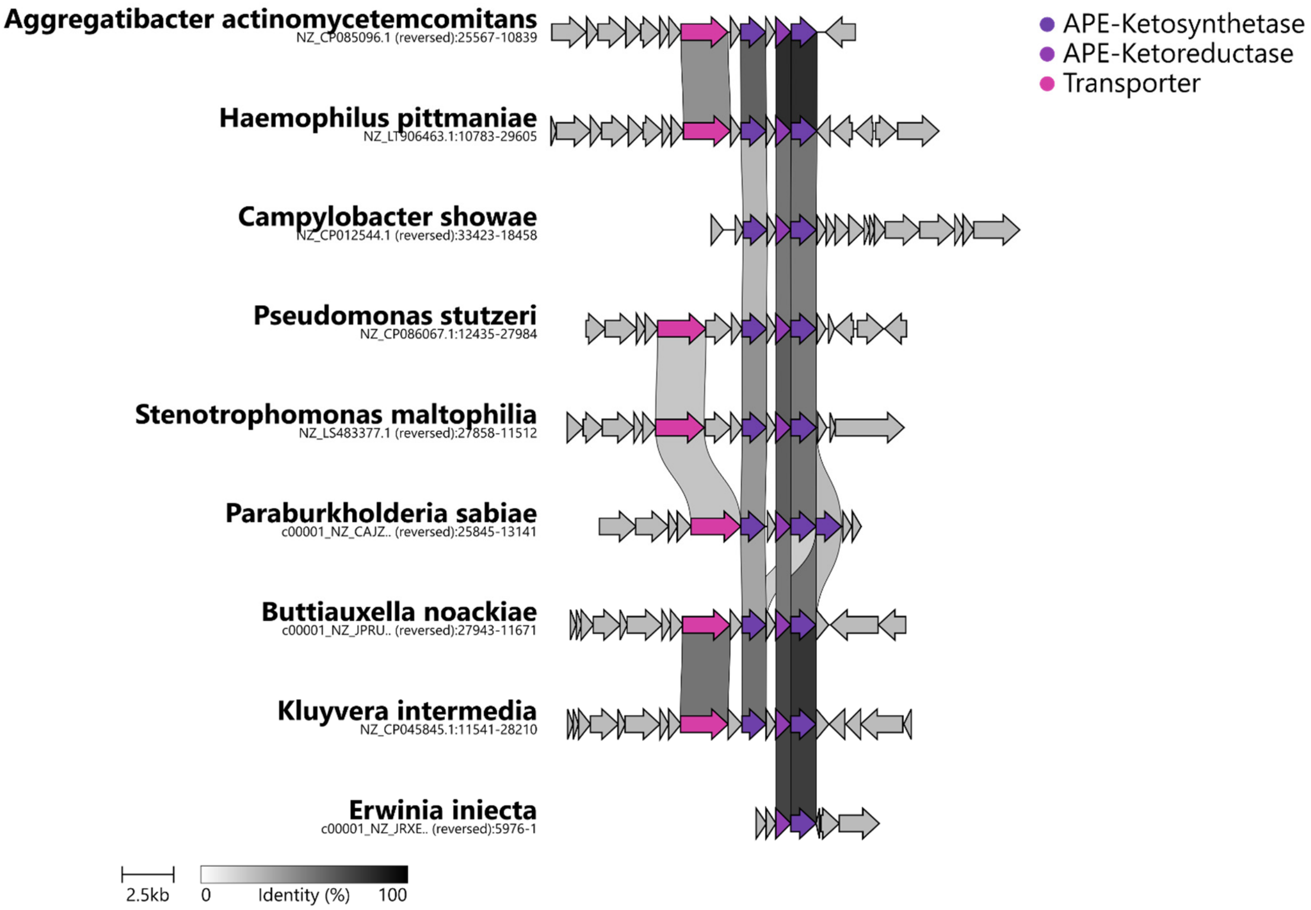

Figure S2: Gene cluster comparison of different exemplary arylpolyene geneclusters from our analysis. Genes within a gene cluster are color-coordinated. The links between the genes show their similiarity.

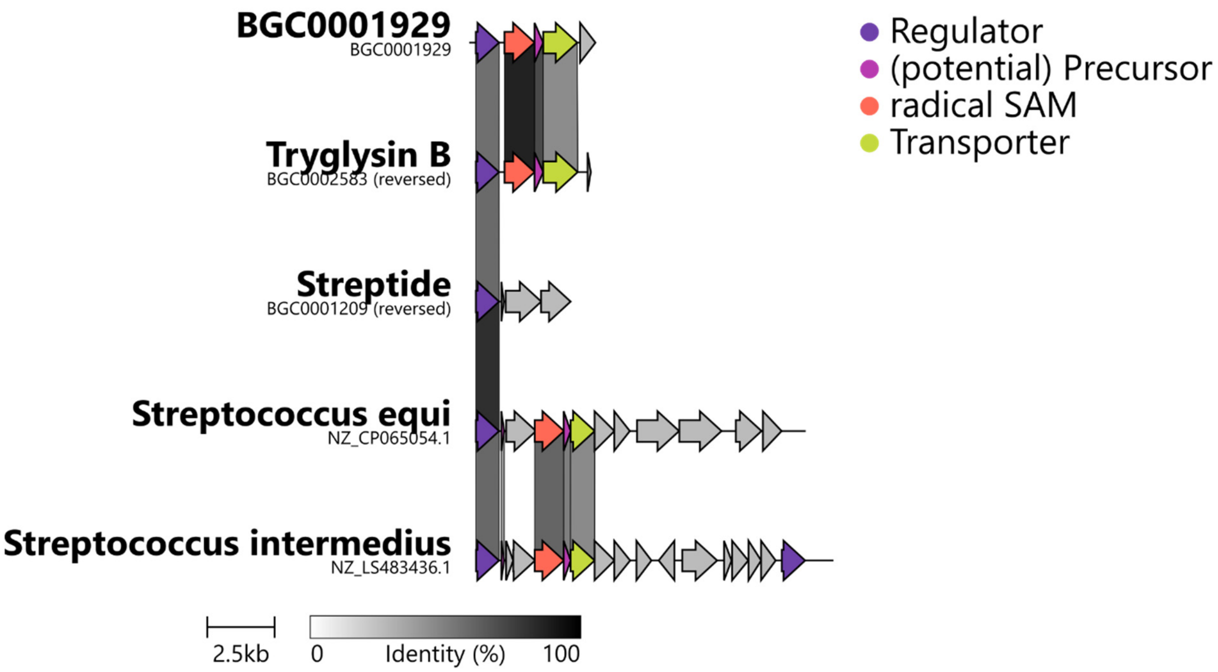

Figure S3: Gene cluster comparison of different RaS-RiPP geneclusters within the genus *Streptococcus* and MIBiG reference clusters. Genes within a gene cluster are color-coordinated. The links between the genes show their similiarity.

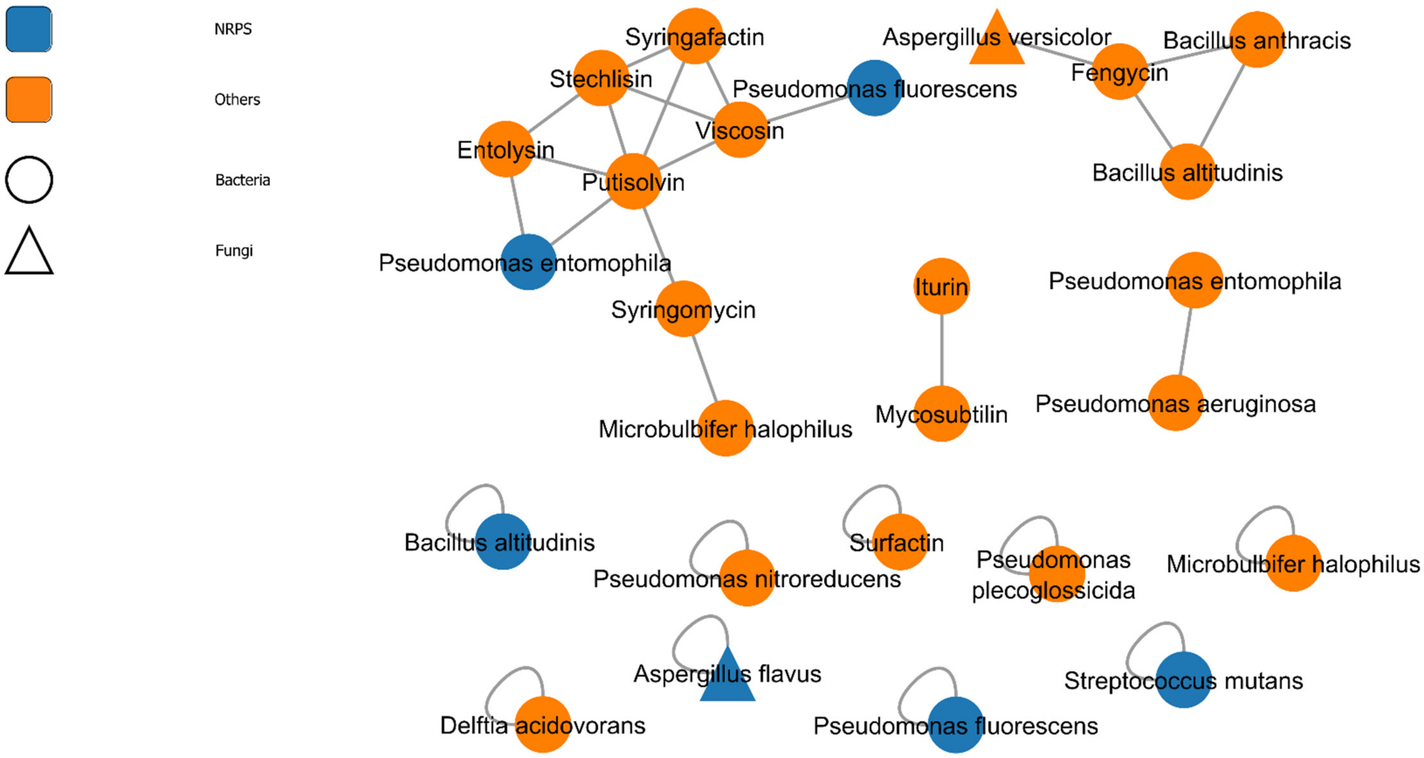

Figure S4: BiG-SCAPE sequence similarity network (SSN) of different lipopeptide geneclusters and MIBiG reference clusters. The network files used were obtained after BiG-SCAPE analysis with a cutoff value of 0.7 and the mix parameter. Subsequent visualization was performed using Cytoscape. Different colors represent classes of natural products; shapes indicate the domain of life of the organism. Gene clusters from our database are labeled with the name of the according organism while MIBiG reference clusters are labeled with the name of the according compound.

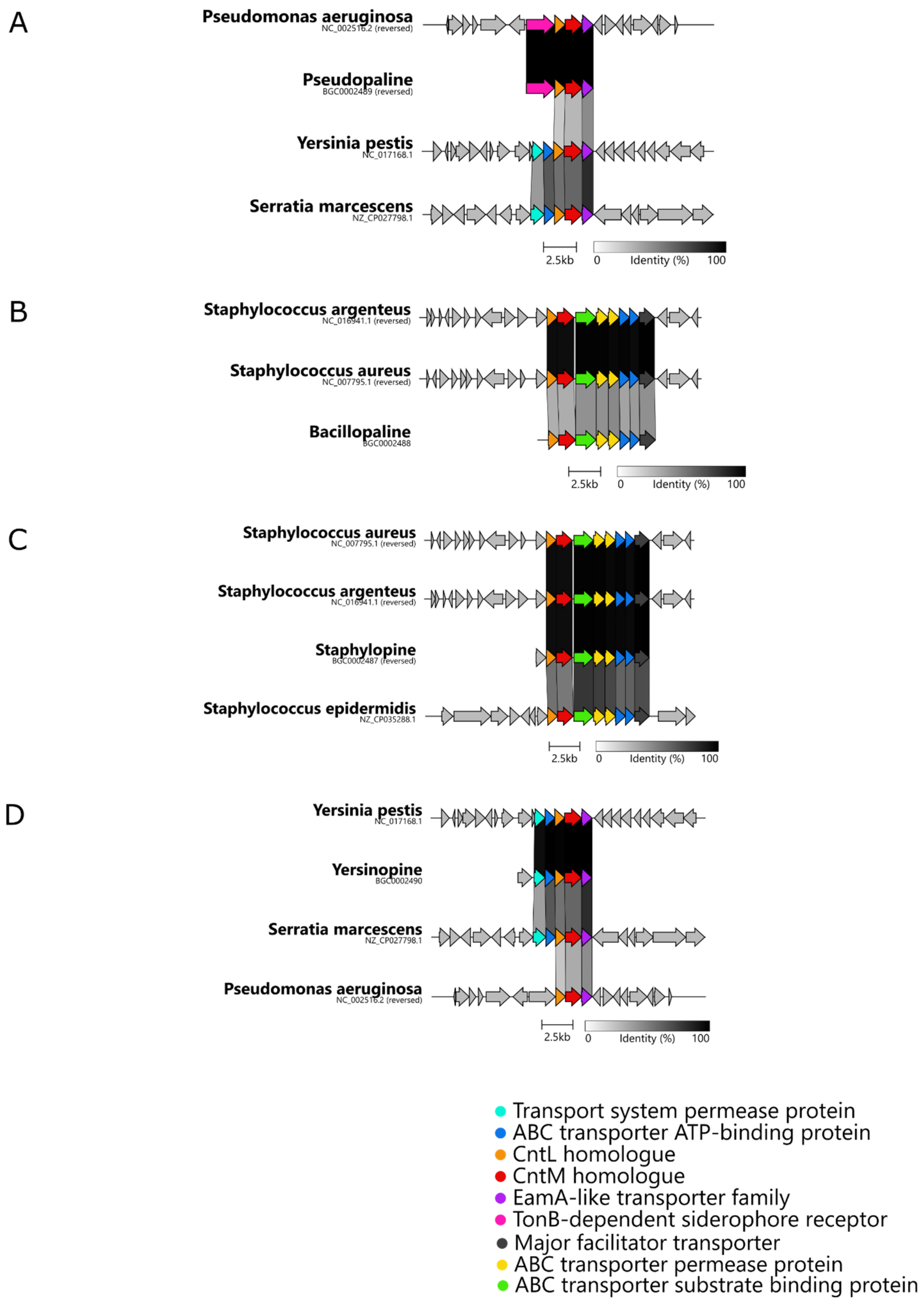

Figure S5: Gene cluster comparison of different zincophore geneclusters and MIBiG reference clusters: (a) Biosynthetic gene clusters of the pseudopaline type; (b) Biosynthetic gene clusters of the bacillopaline type; (c) Biosynthetic gene clusters of the staphylopine type; (d) Biosynthetic gene clusters of the yersinopine type. Genes within a gene cluster are color-coordinated. The links between the genes show their similiarity.

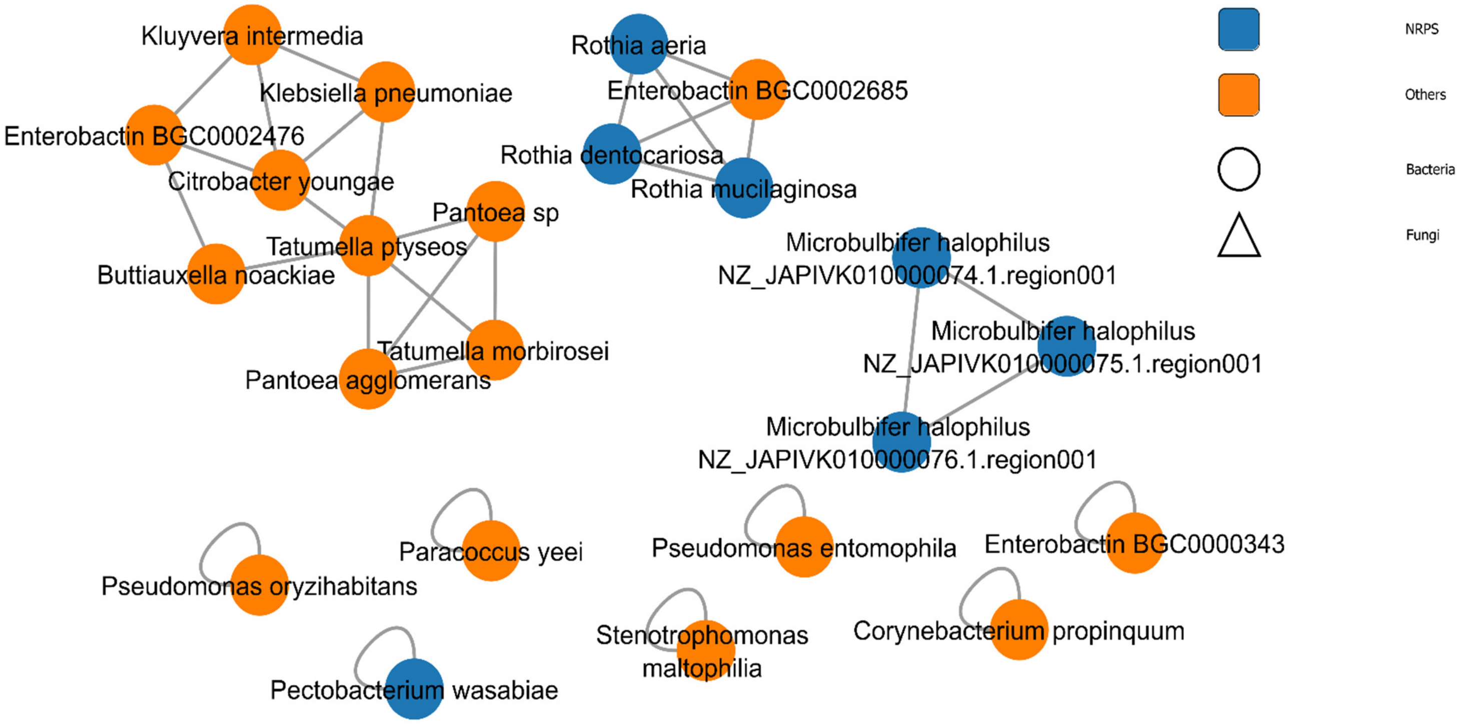

Figure S6: BiG-SCAPE sequence similarity network (SSN) of different siderophore geneclusters from the enterobactin group and MIBiG reference clusters. The network files used were obtained after BiG-SCAPE analysis with a cutoff value of 0.6 and the mix parameter. Subsequent visualization was performed using Cytoscape. Different colors represent classes of natural products; shapes indicate the domain of life of the organism. Gene clusters from our database are labeled with the name of the according organism while MIBiG reference clusters are labeled with the name of the according compound.
